# Association of diabetes type and chronic diabetes complications with early exit from the labour force: register-based study of people with diabetes in Finland

**DOI:** 10.1007/s00125-020-05363-6

**Published:** 2021-01-21

**Authors:** Olli Kurkela, Leena Forma, Pirjo Ilanne-Parikka, Jaakko Nevalainen, Pekka Rissanen

**Affiliations:** 1 Health Sciences, Faculty of Social Sciences, Tampere University, Tampere, Finland; 2 Finnish Institute for Health and Welfare, Helsinki, Finland; 3 Faculty of Social Sciences, University of Helsinki, Helsinki, Finland; 4 Finnish Diabetes Association, Tampere, Finland

**Keywords:** Complications, Early exit from the labour force, Productivity costs, Register study, Type 1 diabetes, Type 2 diabetes

## Abstract

**Aims/hypothesis:**

Diabetes and diabetes complications are a cause of substantial morbidity, resulting in early exits from the labour force and lost productivity. The aim of this study was to examine differences in early exits between people with type 1 and 2 diabetes and to assess the role of chronic diabetes complications on early exit. We also estimated the economic burden of lost productivity due to early exits.

**Methods:**

People of working age (age 17–64) with diabetes in 1998–2011 in Finland were detected using national registers (*N*_type 1_ = 45,756, *N*_type 2_ = 299,931). For the open cohort, data on pensions and deaths, healthcare usage, medications and basic demographics were collected from the registers. The outcome of the study was early exit from the labour force defined as pension other than old age pension beginning before age 65, or death before age 65. We analysed the early exit outcome and its risk factors using the Kaplan–Meier method and extended Cox regression models. We fitted linear regression models to investigate the risk factors of lost working years and productivity costs among people with early exit.

**Results:**

The difference in median age at early exit from the labour force between type 1 (54.0) and type 2 (58.3) diabetes groups was 4.3 years. The risk of early exit among people with type 1 diabetes increased faster after age 40 compared with people with type 2 diabetes. Each of the diabetes complications was associated with an increase in the hazard of early exit regardless of diabetes type compared with people without the complication, with eye-related complications as an exception. Diabetes complications partly but not completely explained the difference between diabetes types. The mean lost working years was 6.0 years greater in the type 1 diabetes group than in the type 2 diabetes group among people with early exit. Mean productivity costs of people with type 1 diabetes and early exit were found to be 1.4-fold greater compared with people with type 2 diabetes. The total productivity costs of incidences of early exits in the type 2 diabetes group were notably higher compared with the type 1 group during the time period (€14,400 million, €2800 million).

**Conclusions/interpretation:**

We found a marked difference in the patterns of risk of early exit between people with type 1 and type 2 diabetes. The difference was largest close to statutory retirement age. On average, exits in the type 1 diabetes group occurred at an earlier age and resulted in higher mean lost working years and mean productivity costs. The potential of prevention, timely diagnosis and management of diabetes is substantial in terms of avoiding reductions in individual well-being and productivity.

**Graphical abstract:**

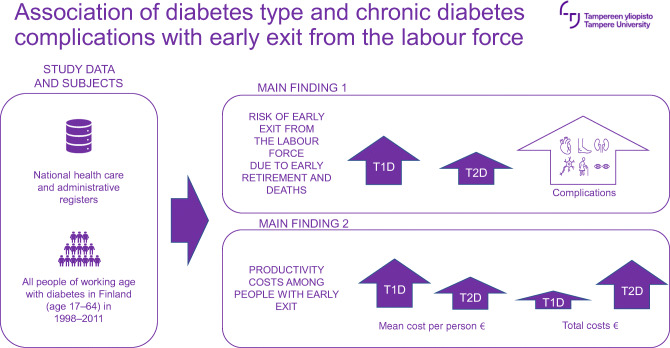

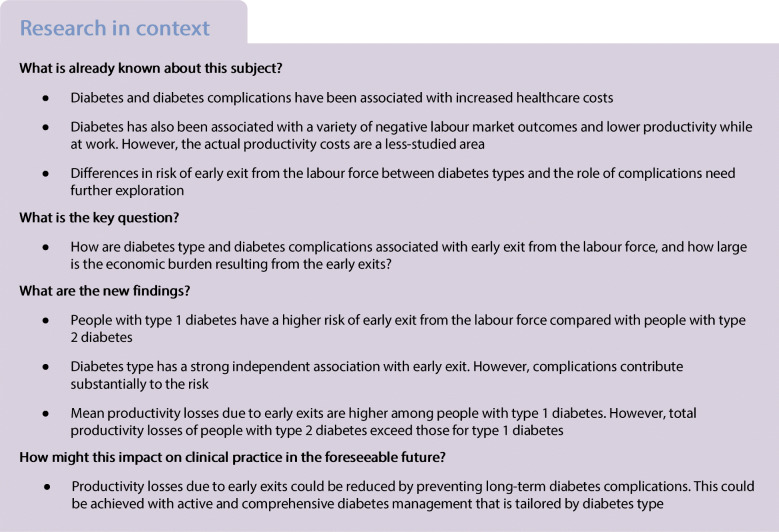

**Supplementary Information:**

The online version of this article (10.1007/s00125-020-05363-6) contains peer-reviewed but unedited supplementary material.

## Introduction

An increase in the incidence of type 1 diabetes has been observed all over the world, and Finland is one of the countries with the strongest trend [1, 2]. Simultaneously, the prevalence of type 2 diabetes has demonstrated steady growth globally and in Finland [1, 3]. Recent estimates from a Finnish health survey state that the prevalence of diabetes among adults over 30 years of age was 13% among men and 9% among women [4]. The majority of cases are of type 2 diabetes (85%) [3]. Since the factors behind type 2 diabetes are better known, cost-effective means to prevent and manage the disease have also been developed [5].

Various cost studies have associated both type 1 and type 2 diabetes with increased healthcare costs [6]. Diabetes has also been associated with a variety of negative labour market outcomes such as unemployment, sick leave, early retirement, death and lowered on-the-job productivity. Type 1 diabetes is associated with higher healthcare expenditure per person compared with type 2 [6], but the difference in early exit from the labour force between diabetes types remains unexplored. Typically, studies have either adopted the perspective of one of the diabetes types or considered both types together, and no previous study has compared the risk between type 1 and type 2 [7].

Delayed diagnosis or inadequate treatment of diabetes can result in complications with varying levels of disability [1]. Globally, the prevalence of severe diabetes complications such as acute myocardial infarction, stroke and lower extremity amputation has been shown to have decreased in the past few decades, especially among people with type 2 diabetes. A similar trend has been observed in Finland. However, the prevalence of multiple complications among people with type 1 diabetes has been shown to have increased [8, 9]. Diabetes complications have been shown to increase healthcare costs substantially [10–12], but the evidence of association of diabetes complications with early exit from the labour force is scarce [13, 14].

Impaired ability to participate in the labour force can result in a substantial reduction in quality of life and increased productivity losses, which have consequences for both individuals and society [15, 16]. The economic burden related to productivity losses caused by morbidity is typically referred to as productivity costs [17]. As onset of both type 1 and type 2 diabetes typically occurs during working age [1], diabetes is a notable cause of productivity costs [6]. Diabetes cost studies estimating productivity costs have typically been carried out with cross-sectional designs, which do not allow consideration of temporal patterns of early exits [6].

The study aims were: (1) to examine the differences in early exits from the labour force between people with type 1 and 2 diabetes; (2) to assess the role of chronic diabetes complications in early exit; and (3) to estimate the mean lost working years and productivity costs related to early exits. We characterised the risk of early exit and the association of complications with the risk among people of working age with diabetes in Finland, and distinguished between type 1 and type 2 diabetes to reflect the differences in disease severity and management.

Avoiding impaired ability to work and the resulting reductions in quality of life and income is important to both individuals and society. Knowledge of how the risk of early exit develops with age among the different subtypes of diabetes, and how complications are associated with the risk, is essential to enhance the cost-effectiveness of interventions and disease management strategies. In parallel with increasing the statutory retirement age, effort should be devoted to reducing early exits from the labour force caused by morbidity.

## Methods

**Fig. 1:**
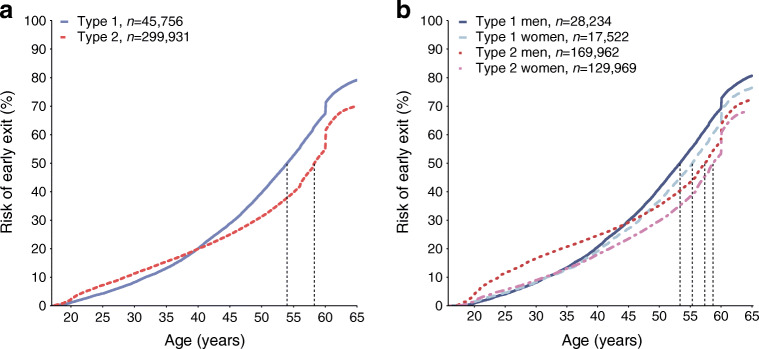
The risk of early exit from the labour force in the study cohort, by diabetes type (**a**) and by sex and diabetes type (**b**). Median ages of early exit are indicated with vertical, dashed lines

Study data originated from national healthcare and administrative registers (Electronic supplementary material [ESM] Fig. 1). Personal identity codes enabled the linkage of information from different registers and the collection of a detailed panel on health service use and pensions [18]. Full details of the cohort were reported by Sund and Koski [3].

### Identification of people with diabetes and diabetes type classification

Study data consist of all people with diagnosed diabetes (excluding people with gestational diabetes) in Finland during 1998–2011 (ESM Fig. 1). A person was included in the study as having diabetes if: (1) a record of entitlement to reimbursement for or a purchase of glucose-lowering medication or insulin was found; or (2) a diabetes diagnosis (ESM Table 1) related to a visit or stay at an inpatient or secondary outpatient service provider was detected; or (3) a diabetes-related entry was found in the Medical Birth Register, Diabetes in Finland (FinDM 1) study [19] or Causes of Death Register. The date of the first entry was considered the date of onset of diabetes. Diabetes type classification was primarily based on data on entitlements for special reimbursement for diabetes-related medication and actual medication purchases (ESM Methods).

### Detection of diabetes complications

Chronic diabetes complications were defined as long-term micro- and macrovascular complications that are likely preventable by proper control of blood glucose and cardiovascular risk factors [20]. Detection of complications was based on a person’s healthcare service use, for which follow-up data were available for a maximum of 14 years (1998–2011) (ESM Fig. 1). Complications were grouped into six categories: kidney-related, eye-related, neuropathic, musculoskeletal, foot and cardiovascular complications. If an entry with complication diagnosis (ESM Table 2) was detected in any of the care registers subsequent but not prior to an entry with diabetes diagnosis, the individual was considered to have a diabetes complication. The earliest entry found was regarded as the date of onset of the complication and the person was classified as having the complication from the date of the first entry until the end of the follow-up.

### Definition of follow-up periods and outcome

Working age was defined as 17–64 years. A person entered the study either on 1 January 1998 (at age between 17–64) or when that person reached working age (17th birthday) (ESM Fig. 2). By allowing for a person’s late entry into the study, study subjects form an open cohort. Age was used as the time scale.

Data on pensions (effective for at least 1 day during 1998–2011) were collected for the cohort (ESM Fig. 1), including the duration and type of the pension. The data were complemented with date of birth, date of death and sex, which was recognised as a confounder.

People that were not considered to be of working age during the study period and people whose pension had started before 1998 were excluded (*n* = 178,466), resulting in 345,687 people with diabetes.

Each person was followed for a maximum of 14 years until early exit from the labour force occurred or the person was censored. The outcome of early exit was defined as the first of: early retirement due to disability; early retirement due to unemployment; early retirement on a part-time pension; or death before age 65. All exits were considered irreversible. Retirement on an old age pension was considered a natural manner of exiting the labour force, and these people were treated as censored. In addition, people that turned 65 or reached the end of the study (31 December 2011) without any of the outcomes occurring were censored.

### Assessment of productivity costs

Lost productivity included losses due to early retirement and death before age 65. Individual lost working years for people that had retired prematurely during 1998–2011 were estimated by calculating the difference between the day when the person reached age 65 and the day of early exit. The sensitivity of productivity costs on assumption of retirement age was analysed using the average age of retirement in the Finnish population in 2011 (60 years) [21] as an alternative to the age of 65.

Productivity costs were estimated using individual-level annual income data (ESM Fig. 1). Since people with diabetes are prone to productivity losses already before retirement [14], we calculated the median of annual incomes from the last 5 years before the exit for each individual and used it as an estimate for productivity cost. For people without income information (*n* = 368), diabetes type- and sex-specific median incomes were used.

### Statistical methods

The risk of early exit from the labour force was analysed using the Kaplan–Meier method. Late entry to the risk set was adjusted for. Survival curves were presented separately for type 1 and type 2 diabetes groups and by sex among these groups.

The association of diabetes type and complications with the hazard of early exit from the labour force was assessed using extended Cox regression models with time-dependent covariates. The first model estimated the crude, overall relative difference in the hazard between diabetes types, regardless of when diabetes was diagnosed. The second model adjusted the hazard ratio for time-dependent complications and sex. As a lack of proportionality was noticed in a visual inspection of the hazards, the third model examined the association of type 1 and type 2 diabetes onsets with early exit, modelled as dichotomous time-dependent covariates. In the fourth model, the results of this model were adjusted for time-dependent complications and sex.

Associations of the risk factors with lost working years and productivity costs were investigated with linear regression analysis. The first model examined the association of diabetes type with lost working years. The second model adjusted the model by complications and sex. The third and fourth models implemented the same strategy on productivity costs.

Statistical significance was set at a 5% level. Analyses were carried out using R statistical software 3.6.0 (‘survival’ package) [22].

## Results

### Population characteristics

The study cohort consisted of 345,687 people with diabetes. The majority (87%) had type 2 diabetes and 57% were men (Table 1).

**Table 1:** Characteristics of the study population according to diabetes type

Characteristic	Type 1 diabetes *n* (%)	Type 2 diabetes *n* (%)
*N*	45,756	299,931
Mean age at diabetes onset (mean ± SD)	29 ± 19	55 ± 11
Sex		
Men	28,234 (61.7)	169,962 (56.7)
Women	17,522 (38.3)	129,969 (43.3)
People with complications	21,417 (46.8)	48,566 (16.2)
Kidney	4705 (10.3)	4830 (1.6)
Eye	15,083 (33.0)	10,858 (3.6)
Neuropathic	3269 (7.1)	4235 (1.4)
Musculoskeletal	3494 (7.6)	5324 (1.8)
Foot	2613 (5.7)	3245 (1.1)
Cardiovascular	7056 (15.4)	33,700 (11.2)
People according to the number of complications		
0	24,339 (53.2)	251,365 (83.8)
1	12,269 (26.8)	38,398 (12.8)
2	5402 (11.8)	7597 (2.5)
3 or more	3746 (8.2)	2571 (0.9)
People with different endpoints		
Censored	32,299 (70.6)	199,390 (66.5)
Pensions	9663 (21.1)	89,636 (29.9)
Death	3794 (8.3)	10,905 (3.6)

People with complications are presented as number of people with at least that specific complication

Complications were more common among people with type 1 diabetes: nearly 50% were diagnosed with complications from one or more complication categories, while among people with type 2 diabetes this was fewer than 20%. In the type 1 diabetes group, almost 10% were diagnosed with complications from three or more complication categories, while in the type 2 diabetes group this was fewer than 1%.

In the type 1 diabetes group, over 30% were diagnosed with at least one eye-related complication. Eye-related complications were the most common complication group in the type 1 diabetes group, before cardiovascular complications. In the type 2 diabetes group, cardiovascular complications were most frequent, with over 10% of people diagnosed with one or more complications from this category. Over 5% with type 1 diabetes and 1% with type 2 were diagnosed with diabetes foot complications, making it the least frequent complication group in both diabetes type groups. Complications were more common in older age groups, with the exception of eye-related complications in the type 1 diabetes group (ESM Table 3).

The relative frequency of early exit from the labour force due to early retirement was higher in the type 2 diabetes group compared with the type 1 diabetes group. In the type 1 diabetes group, the relative frequency of deaths before age 65 was higher compared with the type 2 diabetes group.

### Risk of early exit from the labour force

The difference in median age at early exit from the labour force between the type 1 (54.0 years; 95% CI 53.7, 54.3) and type 2 (58.3 years; CI 58.2, 58.4) diabetes groups was 4.3 years (Fig. 1a). In early adulthood, the risk of early exit in the type 2 diabetes group was higher compared with the type 1 diabetes group. However, the risk of early exit increased more rapidly in the type 1 diabetes group and exceeded that of the type 2 diabetes group at age 40. The risk of early exit by 55 years of age was 53% and 40% for people with type 1 and type 2 diabetes, respectively. The difference in risk of early exit was highest at age 56 (13.4%) and remained similar until age 60. The sudden increase in risks of early exit at age 60 is due to the possibility of retiring on an unemployment pension.

Close to the statutory retirement age, men had a notably higher risk of early exit than women in both diabetes type groups. Among men, the median age at early exit was 53.3 (CI 52.9, 53.7) and 57.4 (CI 57.0, 57.8) for people with type 1 diabetes and type 2 diabetes, respectively, resulting in a difference of 4.1 years (Fig. 1b). Among women, the difference was 3.4 years (type 1: 55.3 [CI 54.9, 55.8]; type 2: 58.7 [CI 58.5, 58.9]). Men with type 2 diabetes had a notably higher risk of early exit in early adulthood compared with all other groups. The risk for both men and women with type 1 diabetes exceeded that of men with type 2 diabetes approximately at age 47. When only people with an early exit were considered, women had a higher risk of early exit in both diabetes type groups throughout the working age.

### Association of diabetes type and complications with early exit from the labour force

According to the crude comparison, the hazard of early exit from the labour force was 28% lower for people with type 2 diabetes compared with type 1 (Model 1, Table 2). When the onset of diabetes was controlled for, type 1 and type 2 diabetes were observed to increase the hazard by 62% and 18%, respectively, compared with those without a diagnosis at the same age (Model 3).

**Table 2:** Hazard ratios for diabetes type, complications and sex, for early exit from the labour force

Risk factor	HR (95% CI)			
	Model 1^a^	Model 2^b^	Model 3^c^	Model 4^d^
Diabetes type^e^	0.72 (0.71, 0.74)	0.87 (0.85, 0.88)		
Diabetes onset type 1^f^			1.62 (1.58, 1.65)	1.22 (1.19, 1.25)
Diabetes onset type 2^g^			1.18 (1.17, 1.20)	1.04 (1.02, 1.05)
Complications^h^				
Kidney		1.71 (1.66, 1.77)		1.70 (1.64, 1.76)
Eye		1.01 (0.99, 1.04)		1.00 (0.97, 1.02)
Neuropathic		1.32 (1.27, 1.38)		1.32 (1.27, 1.37)
Musculoskeletal		1.32 (1.27, 1.37)		1.30 (1.25, 1.35)
Foot		1.28 (1.22, 1.33)		1.28 (1.22, 1.33)
Cardiovascular		1.79 (1.76, 1.83)		1.76 (1.73, 1.80)
Sex^i^		1.02 (1.01, 1.03)		1.02 (1.01, 1.03)

^a^Diabetes type as an overall grouping factor, unadjusted

^b^Relative difference in hazard between diabetes types, adjusted

^c^Diabetes onsets as time-dependent factors, unadjusted

^d^Association of type 1 and type 2 time-dependent diabetes onsets with hazard, adjusted

^e^Reference: type 1

^f^Reference: no type 1 diabetes, time-dependent

^g^Reference: no type 2 diabetes, time-dependent

^h^Reference: no particular complication

^i^Reference: women

After inclusion of complications in the models, the hazard ratios approached unity between diabetes types, approximately to one-half of the unadjusted ratio. Complications were observed to increase the hazard (Model 2), with eye-related complications as an exception. Cardiovascular complications demonstrated the largest effect, increasing the hazard nearly 80%, followed by kidney-related complications (Model 2). Hazard ratios of the complications differed only slightly when examined together with the overall grouping or the time-dependent diabetes onsets (Models 2 and 4). Men had a slightly higher hazard compared with women.

### Productivity costs

On average, type 1 diabetes was associated with more lost working years and higher productivity costs than type 2 diabetes (Table 3, ESM Table 4). Direct association with productivity costs was observed for all complication categories, with eye-related complications as an exception. Out of the most common complication combinations, co-existent cardiovascular and kidney complications were associated with the highest mean lost working years and productivity costs in both diabetes types and regardless of sex (Table 3).

**Table 3:** Mean lost working years and productivity costs among people with the ten most typical complication combinations (in descending order)

Complication status	*n*		Lost working years (mean)		Productivity costs (mean)	
	Type 1	Type 2	Type 1	Type 2	Type 1	Type 2
Overall	45,756	299,933	3.9	2.1	56,877	37,161
Men						
No complications	15,449	137,377	3.6	2.0	53,486	40,050
Cardiovascular	1551	17,877	4.2	2.7	67,586	54,150
Eye	4289	3391	3.4	1.9	53,958	40,522
Musculoskeletal	336	1278	4.1	2.5	71,620	58,184
Cardiovascular & eye	801	1528	4.1	2.6	68,059	54,623
Kidney	484	1202	5.6	4.0	82,334	68,898
Neuropathic	497	1166	4.9	3.4	75,994	62,558
Cardiovascular & kidney	227	953	6.2	4.7	96,434	82,998
Eye & kidney	706	256	5.4	3.9	82,806	69,370
Eye & musculoskeletal	359	131	3.9	2.4	72,092	58,656
Cardiovascular & neuropathic	137	689	5.5	4.0	90,094	76,658
Women						
No complications	8890	113,988	3.2	1.7	36,347	22,911
Cardiovascular	708	7598	3.9	2.3	50,447	37,011
Eye	3199	2277	3.1	1.6	36,819	23,383
Musculoskeletal	471	1919	3.7	2.2	54,480	41,044
Cardiovascular & eye	497	730	3.7	2.2	50,919	37,483
Kidney	290	543	5.2	3.7	65,195	51,758
Neuropathic	200	458	4.5	3.0	58,855	45,419
Cardiovascular & kidney	97	291	5.9	4.3	79,295	65,859
Eye & kidney	490	110	5.1	3.6	65,667	52,231
Eye & musculoskeletal	585	149	3.6	2.1	54,953	41,517
Cardiovascular & neuropathic	43	197	5.2	3.7	72,955	59,519

Means were estimated by the linear regression model

Lengths of CIs (not shown) ranged from 0.05 to 0.32 and from 992 to 6709 for lost working years and productivity costs, respectively

Mean lost working years before age 65 were estimated for people who had exited the labour force early in 1998–2011 (Table 4). The difference in mean lost working years between the type 1 and type 2 diabetes groups was 6.0 years, in favour of type 2 diabetes. Estimates were sensitive to assumption of expected age of retirement. In the type 1 diabetes group, a difference of 2.3 years (€35,895) was observed when comparing the estimates resulting from different expected ages of retirement (65 and 60 years). In the type 2 diabetes group, the difference was 1.7 years (€28,494).

**Table 4:** Lost working years (mean ± SD) and productivity costs (mean ± SD in 2011 in euros) among people that exited the labour force early in 1998–2011

	*n*		Lost working years (mean ± SD)		Productivity costs (mean ± SD)	
	Type 1	Type 2	Type 1	Type 2	Type 1	Type 2
Scenario 1^a^						
Overall	13,457	100,541	14.0 ± 10.3	8.0 ± 6.7	206,568 ± 215,432	143,689 ± 163,680
Sex						
Men	8898 (66.1)	59,728 (59.4)	13.9 ± 10.0	8.0 ± 6.5	219,470 ± 233,440	160,522 ± 185,508
Women	4559 (33.9)	40,813 (40.6)	14.1 ± 10.9	8.1 ± 7.0	181,383 ± 169,704	119,054 ± 120,893
Scenario 2^b^						
Overall	10,847	61,779	11.7 ± 9.7	6.3 ± 6.8	170,673 ± 185,500	115,195 ± 137,782
Sex						
Men	7215 (66.5)	36,952 (59.8)	11.5 ± 9.4	6.2 ± 6.5	178,021 ± 200,536	125,632 ± 152,131
Women	3632 (33.5)	24,827 (40.2)	12.1 ± 10.3	6.5 ± 7.2	156,073 ± 150,214	99,661 ± 111,309

Data for men and women with type 1 and type 2 diabetes are given as *n* (%)

^a^Scenario 1: expected age of retirement 65

^b^Scenario 2: expected age of retirement 60

Among people with early exit, median annual income from the last 5 years before early exit in type 1 and 2 diabetes groups was €14,318 and €17,558, respectively. In the type 1 diabetes group, mean productivity costs were 1.4-fold compared with the type 2 diabetes group (Table 4). Total productivity costs of people with early exit were €2800 million and €14,400 billion for people with type 1 and type 2 diabetes, respectively. On average, women lost more working years than men in both diabetes groups, but the differences were minor (type 1: 0.2 years, €38,087; type 2: 0.1 years, €41,468).

## Discussion

We examined differences in early exit from the labour force between people with type 1 and 2 diabetes, assessed the role of chronic diabetes complications in early exit and estimated the economic burden of lost productivity due to early exits.

We demonstrated a marked difference in the risk of early exit between people with type 1 and type 2 diabetes. The pattern of early exit differed between diabetes type groups: the difference was largest close to statutory retirement age. Diabetes onset of both types resulted in a notable escalation of hazard compared with those remaining without diagnosis at the same age. The escalation was over threefold for type 1 diabetes compared with type 2 diabetes.

People with type 2 diabetes had higher risk of early exit in early adulthood compared with those with type 1 diabetes. This is unexpected, since people with type 1 diabetes have typically managed with insulin dependency longer and all investigated complications were more common in the type 1 diabetes group also in early adulthood. A partial explanation could be that the analyses did not adjust for depression, which is one of the leading causes for disability pensions in Finland [23]. We focused on long-term complications likely caused by diabetes and on those that can be avoided by proper diabetes treatment including support and education.

Overall, chronic diabetes complications were more common in the type 1 diabetes group than in the type 2 diabetes group in all age groups. Our results demonstrated a consistent, increasing hazard of early exit from the labour force in the diabetes cohort for each of the complication categories, with eye-related complications as an exception. Adjustment for complications partially reduced the association of diabetes type (and onset of diabetes) with early exit.

A discrepancy exists in whether or not reported productivity losses are actually attributable to diabetes itself or to its complications. Ervasti et al. demonstrated the association of diabetes complications, age, sex and education with risk of work disability [14]. Rodríguez-Sánchez and Cantarero-Prieto suggested that the negative effect of diabetes on unemployment and lower income is largely mediated by various factors, including a person’s age and sex, complications and lifestyle factors [13]. However, two Finnish studies have found that early exit from the labour force among people with diabetes differs only slightly from the Finnish population [24, 25]. These studies did not distinguish between diabetes types or assess the role of complications. Our study associated productivity losses primarily with diabetes complications but also pointed out the notable association of diabetes onset with early exit in both diabetes type groups. Per person mean productivity costs were nearly twofold in the type 1 diabetes group compared with the type 2 diabetes group among all people at working age with diabetes. Each one of the complication categories was associated with an increase in productivity costs. An increase in the number of complications was associated with an increase in productivity costs.

Among those with early exit, people with type 1 diabetes exited the labour force earlier than people with type 2. This resulted in higher mean lost working years and productivity costs compared with people with type 2 diabetes. Mean lost working years among people with type 1 diabetes were substantial, as they covered approximately 30% of the working age.

Median annual income among people with early exit was substantially lower compared with median annual income among the working population in Finland in 2011 (€33,288) [26]. The difference cannot be explained by different methods of calculation alone. People with diabetes tend to have longer unemployment periods and lower income compared with people without diabetes [13]. Diabetes is already associated with work loss before onset of the disease and especially after the onset [14]. A major strength of our estimate of the productivity cost is that it captures individual variability in productivity.

Despite lower mean lost working years among people with type 2 diabetes, the total productivity losses were substantially higher due to its higher prevalence. When the estimated productivity costs were distributed evenly in the study time period, the incidence of early exits between 1998 and 2011 resulted in an annual cost of approximately €1200 million, which constituted as much as 0.5% of the gross domestic product (GDP) in Finland in 2011 [27]. At the same time, diabetes-related productivity costs, including all early exits and reimbursed absences, were estimated to be threefold compared with diabetes-related healthcare costs (€2552 million vs €832 million) [28]. Estimates of productivity costs were sensitive to assumptions of ages of retirement but remained substantial in all analyses.

Sex was introduced in the analyses as a confounder, but it had only a minor role in the risk of early exit. However, among early exits, women had on average lower productivity costs even though the lost working years were not found to be markedly different. This is possibly due to lower average income among women.

A major strength of the study is that it captures all people of working age with diabetes during 1998–2011 in Finland. Thus, selection bias was minimised and estimates were highly generalisable on a national level. Furthermore, data were based on high-quality registers [29] with high volume and a long follow-up period, which enabled reliable estimation of onsets of complications and labour market outcomes. Studies with longitudinal designs are scarce [14, 24, 30, 31]. We could estimate the development of risk of early exit at different stages of working life and demonstrate differences in early exit patterns among diabetes type groups.

It was not possible to account for depression and social deprivation, which is a limitation of the study. Both impact health behaviour, treatment adherence and outcomes, and working ability, and are important to account for in clinical practice [32, 33]. Furthermore, our study did not consider sick leave and reductions in on-the-job productivity and therefore represents only part of the total productivity costs. According to Jarvala et al, sick leave (over 9 days) contributed 4% of total productivity costs of people with diabetes in Finland in 2007 [34]. Reductions in on-the-job productivity can be relatively large prior to actual absences from work among people with type 2 diabetes [35].

Our study clearly demonstrates the differences between people with type 1 and type 2 diabetes and the notable role of diabetes complications in early exit from the labour force. As diabetes is a major public health issue in Finland and globally [1, 4], its effect on individual well-being and the economy is substantial. Although type 1 diabetes results in relatively more early exits, the majority of economic loss is brought about by type 2 diabetes due to its higher prevalence. As the increase in the prevalence of diabetes is predicted to continue in the future [1], active and comprehensive management of diabetes as well as cardiovascular risk factors to prevent complications is important, not only to decrease the disease burden of individuals and families with diabetes, but also to reduce economic burden related to the early exits. Decision makers should be aware of potential cost savings, not only in healthcare but also as avoided productivity losses.

Reduction in productivity losses could be achieved by intensive diabetes care and secondary prevention. Adults with diabetes in Finland have mainly been treated and followed up by primary healthcare teams. Finnish social care and healthcare will be reorganised in the near future [36], and it is crucial to ensure adequate secondary prevention services and education programmes for people with diabetes. Recent developments in both diabetes technology and medications as well as a chronic care model with patient-centred collaborative care are welcomed. A Finnish healthcare register, including primary and secondary diabetes care, is under development. The knowledge base will include the quality, effectiveness and cost of public healthcare and will enable further investigations of factors behind productivity losses and the cost-effectiveness of means to avoid them.

Our study points out interesting topics for future research. The roles of depression and major psychiatric disorders as diabetes comorbidities in early exit from the labour force should be examined. Social deprivation as a major driver of early onset of type 2 diabetes and complications should be addressed. Quantification of absent cost components (short-term and long-term sick leave, and loss of on-the-job productivity) could bring about a deeper understanding of the nature of rising productivity costs and could better guide the targeting of interventions. Future interventions should also be assessed by their effects on productivity.

## Supplementary Information

ESM 1 (PDF 193 kb)

## Data Availability

The data used in this study are available from the Finnish Institute for Health and Welfare, but restrictions apply to the availability of these data. Data are, however, available from the authors upon reasonable request and with permission from the Finnish Institute for Health and Welfare.
