# Supplementary material for: Association of diabetes type and chronic diabetes complications with early exit from the labour force: register-based study of people with diabetes in Finland
Source: Diabetologia. 2021 Jan 21;64(4):795–804. doi: 10.1007/s00125-020-05363-6 (PMC7940158; doi:10.1007/s00125-020-05363-6)
Supplement: Supplementary file 1 — (PDF 193 kb) [file 125_2020_5363_MOESM1_ESM.pdf]

Electronic supplementary material

ESM Methods

Diabetes type classification

Diabetes type classification was mainly based on data on entitlements for special reimbursement of diabetes-related medication and actual medication purchases. This information was considered more reliable in determining diabetes type compared to actual diagnoses in health care registers, since the usage of diabetes diagnoses are known to vary regionally and temporally in Finland [3]. A person with regular purchases of insulin and with more years of insulin purchases than glucose-lowering medication was classified as a person with type 1 diabetes. A person with type 2 diabetes was assumed to have purchases of glucose-lowering medication affecting secretion of pancreatic insulin or have more years with purchases of glucose-lowering medication (Anatomical therapeutic chemical [ATC] class A10B) than purchases of insulin (ATC class A10A). In case when years of insulin and glucose-lowering medication purchases were equal, person was classified as a person with type 1 diabetes if the age at first diabetes entry did not exceed 40 years.

ESM Table 1

	ICD-8	ICD-9	ICD-10
Type 1		250?B	E10, O24.0
Type 2		250?A	E11, O24.1
Other		250?C	E12, E13, O24.2, P70.2
Unspecified	250	250?X	E14, G59.0, G63,2, H28.0, H36.0, I79.2, M14.2, M14.6, N08.3, O24.3, Z83.3

ESM Table 1. ICD codes used in detection of persons with diabetes.

ESM Table 2

Complication category	ICD-10 codes
Kidney	E10.2, E11.2, E12.2, E13.2, E14.2, N08.3, N08.30*-N08.32*, N08.39*, N04.89, N17.0-N17.9, N18.0-18.9, Z49.0-Z49.2, I12, N11, N13.6
Eye	E10.3, E11.3, E12.3, E13.3, E14.3, H28.0*, H33-H35, H36.00*-H36.05*, H36.09*, H40.5, H42.0*, H43, H54
Neuropathic	E10.4, E11.4, E12.4, E13.4, E14.4, G57.1, G57.3, G57.5, G59.0*, G63.2*, G73.0*, G99.0*, H49, I95.1, I95.8, I95.9, N48.4
Diabetes foot	E10.5, E11.5, E12.5, E13.5, E14.5, I73.8*, I73.9*, I79.2*, M14.6*, S91, T93.0, T87.4, T87.5
Musculoskeletal	M14*, M14.2*, G56.0, M75.0, M82.1
Cardiovascular	G45, G47, I11, I13, I15.0, I20- I25, I42.0-I42.5, I42.8, I42.9, I44-I51, I61-I67, I69, I70, I69, I70, I71, I74, I77.8, I77.9, I97.0, I97.1, N28.0, Z95

* indicates diabetes ICD-10 specification

ESM Table 2. ICD codes used in complication detection.

ESM Results

ESM Table 3

	Type 1<br>n (%)	Type 2<br>n (%)
N	45,756	299,931
-29	16,760 (36.6)	16,650 (5.6)
30-44	22,486 (49.1)	78,594 (26.2)
45-64	26,436 (57.8)	277,228 (92.4)
Mean age at diabetes onset (mean, sd)	29 (19)	55 (11)
Persons with complications	21,417 (46.8)	48,566 (16.2)
Kidney	4,705 (10.3)	4,830 (1.6)
-29	985 (5.9)	112 (0.7)
30-44	2,098 (9.3)	586 (0.7)
45-64	2,810 (10.6)	4,830 (6.1)
Eye	15,083(33.0)	10,858 (3.6)
-29	4,278 (25.5)	96 (0.6)
30-44	7,175 (31.9)	893 (1.1)
45-64	8,397 (31.8)	10,379 (3.7)
Neuropathic	3,269 (7.1)	4,235 (1.4)
-29	403 (2.4)	49 (0.3)
30-44	1,160 (5.2)	349 (0.4)
45-64	2,321 (8.8)	4,010 (1.4)
Musculoskeletal	3,494 (7.6)	5,324 (1.8)
-29	239 (1.4)	45 (0.3)
30-44	1,663 (7.4)	593 (0.8)
45-64	2,480 (9.4)	4,989 (1.8)
Foot	2,613 (5.7)	3,245 (1.1)
-29	187 (1.1)	18 (0.1)
30-44	774 (3.4)	180 (0.2)
45-64	2,030 (7.7)	3,152 (1.1)
Cardiovascular	7,056 (15.4)	33,700 (11.2)
-29	301 (1.8)	119 (0.7)
30-44	1571 (7)	1593 (2)
45-64	5,938 (22.5)	32,904 (11.9)

ESM Table 3. *Age-stratified proportions of persons with complications.*

ESM Table 4

	Model 1[a]	Model 2[b]	Model 3[c]	Model 4[d]
Diabetes type[e]	-1.8	-1.5	-18,523	-13,436
	(-1.9, -1.8)	(-1.6, -1.5)	(-20,910, -18,523)	(-14,725, -12,147)
Complications[f]				
Kidney		2.0		28,848
		(1.9, 2.1)		(26,419, 31,277)
Eye		-0.1		472
		(-0.2, 0.0)		(-1,149, 2,093)
Neuropathic		1.3		22,508
		(1.2, 1.4)		(19,815, 25,201)
Musculoskeletal		0.5		18,134
		(0.4, 0.6)		(15,685, 20,582)
Foot		0.9		15,504
		(0.8, 1.1)		(12,415, 18,592)
Cardiovascular		0.6		14,100
		(0.6, 0.7)		(12,863, 15,338)
Gender[g]		0.3		17,139
		(0.3, 0.4)		(16,224, 18,055)

[a] Difference in lost working years between diabetes types; unadjusted
[b] Difference in lost working years between diabetes types, adjusted for complications
[c] Difference in productivity costs between diabetes types; unadjusted
[d] Difference in productivity costs between diabetes types, adjusted for complications
[e] Reference: Type 1 diabetes
[f] Reference: No complications
[g] Reference: Women

ESM Table 4. *Results of the regression of lost working years and productivity costs (2011 euros) on the risk factors among persons of working age with diabetes.*

ESM Fig. 1.

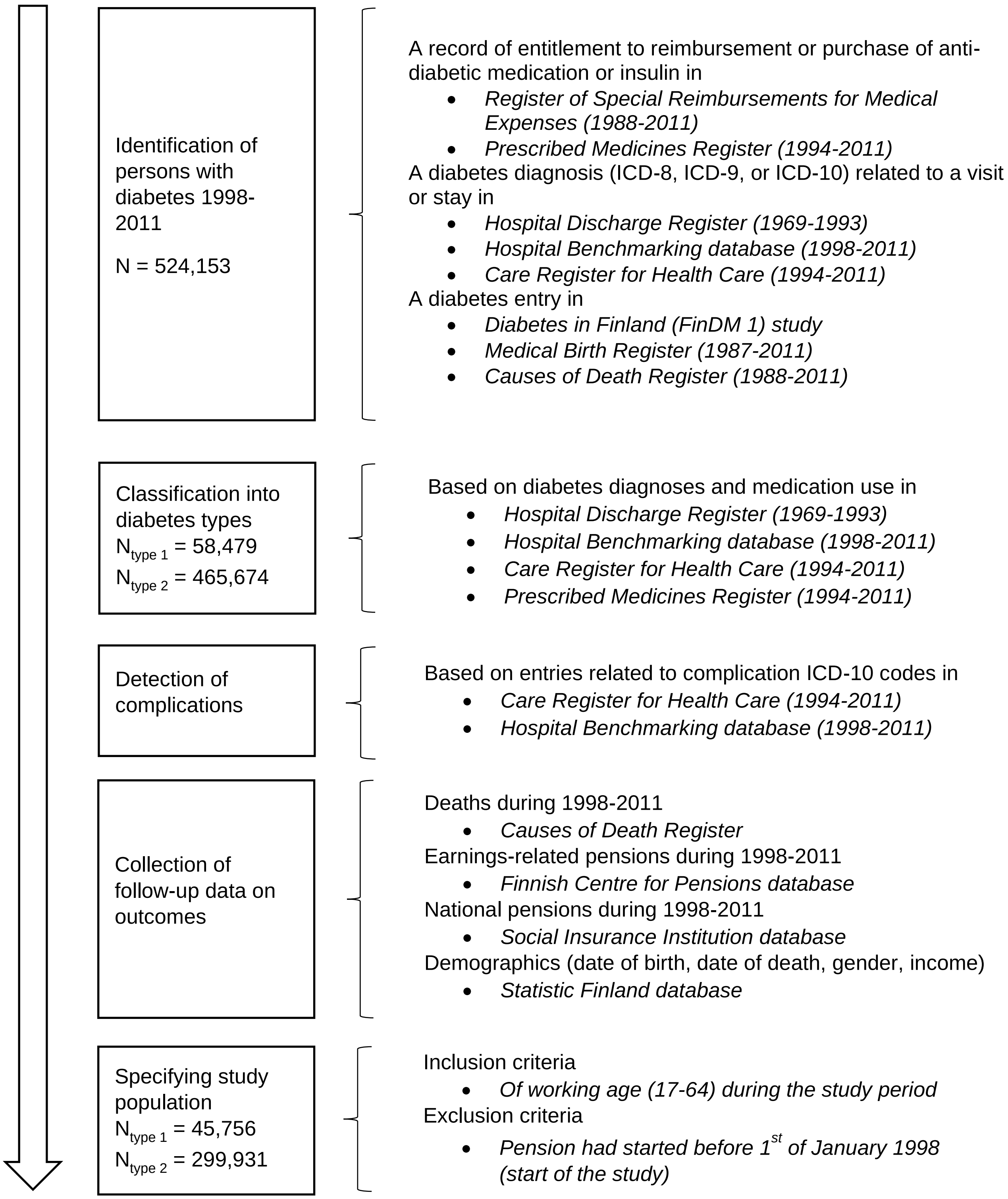

ESM Fig. 1. Detailed list of data sources for diabetes detection, classification into diabetes types, complication detection, and outcomes.

ESM Fig. 2

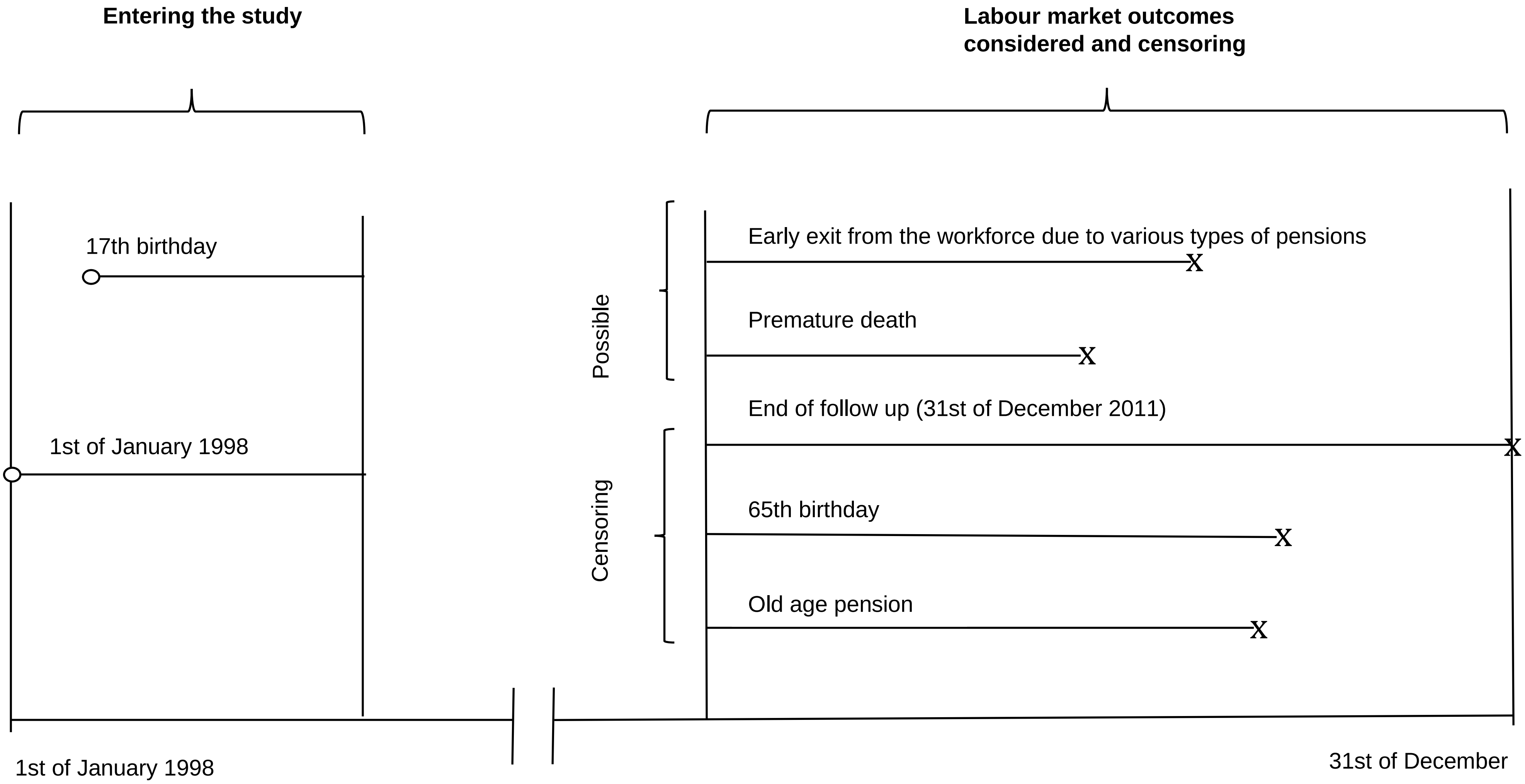

ESM Fig. 2. Study design.
